# Hepatitis C Virus and Natural Compounds: a New Antiviral Approach?

**DOI:** 10.3390/v4102197

**Published:** 2012-10-17

**Authors:** Noémie Calland, Jean Dubuisson, Yves Rouillé, Karin Séron

**Affiliations:** Inserm U1019, CNRS UMR8204, Center for Infection & Immunity of Lille (CIIL), Institut Pasteur de Lille, Université Lille Nord de France, Lille, France; Email: noemie.calland@ibl.fr (N.C.); jean.dubuisson@ibl.fr (J.D.); yves.rouille@ibl.fr (Y.R.)

**Keywords:** hepatitis C virus, natural molecules, direct acting antivirals, flavonoids

## Abstract

Hepatitis C is a major global health burden with an estimated 160 million infected individuals worldwide. This long-term disease evolves slowly, often leading to chronicity and potentially to liver failure. There is no anti-HCV vaccine, and, until recently, the only treatment available, based on pegylated interferon and ribavirin, was partially effective, and had considerable side effects. With recent advances in the understanding of the HCV life cycle, the development of promising direct acting antivirals (DAAs) has been achieved. Their use in combination with the current treatment has led to encouraging results for HCV genotype 1 patients. However, this therapy is quite expensive and will probably not be accessible for all patients worldwide. For this reason, constant efforts are being made to identify new antiviral molecules. Recent reports about natural compounds highlight their antiviral activity against HCV. Here, we aim to review the natural molecules that interfere with the HCV life cycle and discuss their potential use in HCV therapy.

## 1. Introduction

Plants have been used for centuries for the treatment of human diseases. Historically, numerous important modern drugs have been developed from molecules originally isolated from natural sources [1,2]. Among them, the most popular is aspirin, based on a natural product salicin isolated from *Salix alba*. Morphine and codeine were extracted from opium poppy *Papaver somniferum*, and quinine, traditionally used as an anti-malaria treatment, from cinchona tree. In the past decades, taxol, a molecule extracted from the bark of the Pacific yew tree, *Taxus brevifolia,* has become one of the most used anti-cancer agent worldwide [3].

The search for new bioactive molecules in plants in key therapeutic areas such as immunosuppression, infectious diseases, oncology and metabolic diseases is still an active part of pharmaceutical research [4]. About 40 new drugs launched on the market between 2000 and 2010, originate from terrestrial plants, terrestrial microorganisms, marine organisms, and terrestrial vertebrates and invertebrates [5]. The World Health Organization (WHO) estimates that approximately 80% of the world’s population rely mainly on traditional medicine, predominantly originated from plants, for their primary health care. 

Traditional medicines, including Chinese herbal formulations, can serve as the source of potential new drugs. Active plant compounds used either for prophylactic or therapeutic treatments are orally administrated to patients as teas, powders, and other herbal formulations [6,7]. Phenolic compounds are often responsible for the bioactivities of the plant crude extracts. During the last decades, people have tried to identify more precisely the active molecules of these traditional medicines. Another approach was to systematically screen natural molecules present in plant extracts and test the activity of these phytochemicals using the appropriate assays (depending on the pathology studied). 

The main advantage of using natural molecules from plant extracts is a reduced cost of production, with no need of chemical synthesis. This mode of production might lead to less expensive treatments, available for populations of low-income countries. 

Some natural medicines have been shown to possess antiviral activities against herpes simplex virus [8,9], influenza virus, human immunodeficiency virus [10,11,12], hepatitis B and C viruses [13,14]. The screening of natural products has led to the discovery of potent inhibitors of *in vitro* viral growth [15]. Antiviral activities of several hundred natural compounds have been identified worldwide. In addition, dozens of herbs are known to have hepatoprotective activities. During the last decades, scientists have tried to analyze more precisely the active molecules present in this traditional medicine that is frequently used for the treatment of hepatitis in China [16]. 

The last few years have seen a flurry of reports on the identification of natural molecules of plant origin with anti-hepatitis C activities. The aim of this review is to give an overview of these different compounds with a special focus on the most promising molecules.

## 2. Hepatitis C Virus

Hepatitis C is a major healthcare problem worldwide caused by a viral infection with a high tendency to become chronic. Chronic hepatitis C is linked to the development of cirrhosis and hepatocellular carcinoma. The virus responsible for this disease was discovered more than 20 years ago [17]. Its transmission is thought to be essentially parenteral, and has been linked to blood transfusions before its discovery. Since hepatitis C virus (HCV) discovery, blood screening diagnostics have greatly reduced the blood-borne transmission of the virus. However, the transmission still occurs through other modes of contamination and the slow development of the disease results in many persons not knowing their infected status. It is estimated that about 160 million persons (2.35% of the world population) are infected with HCV [18]. 

Currently, there is no vaccine against HCV and the high diversity of viral isolates will probably make it very difficult to develop a vaccine. On the other hand, we know that, in contrast to hepatitis B and human immunodeficiency viruses, HCV can be eradicated from chronically infected patients with antiviral treatments. However, the standard therapy, which is based on a combination of pegylated interferon alpha (IFN-α) and ribavirin [19], results in highly variable outcomes [20], is very expensive and has severe side effects that are difficult to endure for the patients. Nevertheless, it is currently thought that efficient anti-HCV therapies will be achieved with direct acting antivirals (DAAs) [21]. The recent addition of protease inhibitors to the standard anti-HCV therapy has already improved sustained virological response rates in patients infected with genotype 1 HCV. New drugs targeting other viral proteins are in clinical trials and will probably also help improving response to HCV therapy [22,23]. A combination of DAAs will reduce the risk of selecting viral escape mutants. DAAs combinations in the absence of interferon will probably enable to greatly reduce side effects of the therapy, which are mainly associated with the use of interferon and contribute to the failure of the treatment. Ideally, such a combination should include DAAs targeting different steps of the HCV life cycle and should be efficient against all HCV genotypes. Moreover, to have a chance of eradicating HCV, the therapy should be cheap so as to be able to cure infected patients from low-income countries and stop the transmission of the virus.

Hepatitis C virus is a small, enveloped virus belonging to the Hepacivirus genus of the *Flaviviridae* family [24]. Its single-stranded genomic RNA contains a single open reading frame surrounded by two untranslated regions (UTR) that are necessary for the translation and the replication of the viral genome [25,26,27]. The translation of the open reading frame is under the control of an internal ribosome entry site (IRES), located in the 5’UTR. It gives rise to a polyprotein precursor, which is cleaved by host- and viral-encoded proteases into ten polypeptides. The N-terminal part of the polyprotein contains structural proteins: the core protein C, a component of the viral capsid, and the two envelope glycoproteins E1 and E2. The C-terminal part of the polyprotein contains non-structural proteins required for RNA replication: NS3, which has protease and helicase activities; NS4A, a co-factor of NS3 protease; NS4B, a polytopic membrane protein; NS5A, a phosphoprotein; and NS5B, the viral RNA-dependent RNA polymerase. Between the structural proteins and the non-structural proteins involved in RNA replication, the polyprotein also contains two additional polypeptides required for viral assembly, which are dispensable for RNA replication: the viroporin p7 and the NS2 protein, which has an autoprotease activity during the maturation of the polyprotein precursor.

The structure of the viral particle is still unknown. In patients, circulating HCV particles are associated with apolipoproteins (Apo) B and E, and have highly variable buoyant densities, the lighter ones being the most infectious [28]. It is currently thought that infectious HCV particles are initially secreted as very low-density lipoprotein (VLDL)-like particles by infected hepatocytes and then potentially undergo lipolysis in the bloodstream, which progressively converts them into intermediate density lipoprotein (IDL)- and LDL-like particles. However, it is not yet clear what in this process reduces the specific infectivity of HCV viral particles.

Our knowledge of the HCV life cycle has greatly improved in recent years, following the finding of a viral strain (JFH-1) able to replicate in cell culture [29,30,31]. The JFH-1-based cell culture model has been named HCVcc. For reasons that are still unknown, other viral isolates do not efficiently replicate in cell culture. Before the HCVcc model was established, specific steps of the HCV life cycle had been studied with other experimental systems recapitulating RNA replication, with the subgenomic replicon model [32,33], or viral entry, with the HCV pseudoparticles (HCVpp) model [34,35,36]. The replicon model is based on a modified HCV genome, in which the coding region of the structural proteins is replaced by a selection marker. *In vitro* synthesized subgenomic replicon RNA is introduced in cells by electroporation and the cells replicating it express the selection marker and can thus be selected. There is no release of viral particles, and this model only allows studying cellular and molecular mechanisms involved in viral RNA replication. Hepatitis C virus pseudoparticles are retroviral particles pseudotyped with HCV envelope glycoproteins E1E2. In this system, only E1E2-dependent, early entry steps (virus binding, uptake and fusion) are HCV specific, whereas later steps depend on retroviral function.

**Figure 1:**
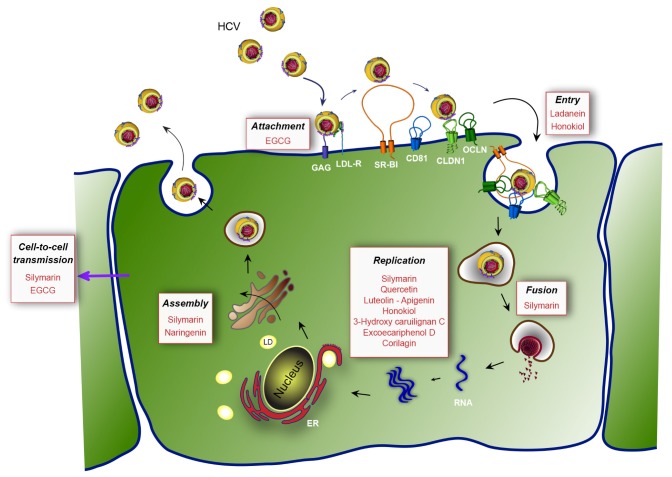
Hepatitis C virus (HCV) life cycle and targets of the most potent natural inhibitors. First, HCV binds to non-specific factors glycosaminoglycans (GAG) and LDL receptor (LDL-R) present at the cell surface (attachment step). Then, the viral particle is directed to specific entry factors (entry step), the scavenger receptor class B type I (SR-BI), the tetraspanin CD81 and the tight junction proteins claudin-1 (CLDN1) and occludin (OCLN). The virus is internalized by endocytosis and the viral genome is released into the cytosol of the cell after fusion with endosomes (fusion step). Next, the translation and the polyprotein processing take place and RNA is replicated (replication step). In the late stages of the cycle, the virion is assembled (assembly step) in the vicinity of cytoplasmic lipid droplets (LD) and is released from the cell. Finally, the released virions can infect adjacent cells by cell-free transmission or cell-to-cell transmission. The affected steps of the viral cycle are in black. The natural compounds are in red. EGCG: epigallocatechin-*3*-gallate; ER: endoplamic reticulum.

The life cycle of HCV can be divided into three major steps: entry of the virus into its target cells by receptor-mediated endocytosis, cytoplasmic and membrane-associated replication of the RNA genome, and assembly and release of the progeny virions (Figure 1). Hepatitis C virus entry is a very complex process, which involves a series of host entry factors [37]. On the viral particle, envelope glycoproteins E1E2 play a major role during entry. The viral particle probably initially binds to glycosaminoglycans (GAG) on the surface of the target cell. It has been proposed that interactions between the LDL receptor (LDL-R) and apolipoproteins of the viral particle might also participate in the initial binding to the cell surface. Following these rather non-specific initial binding events, several host entry factors are specifically involved in the entry process [38]. The tetraspanin CD81 [39], the scavenger receptor class B type I (SR-BI) [40], and the tight junction proteins claudin-1 (CLDN1) [41] and occludin (OCLN) [42,43] are mandatory for HCV entry. Epidermal growth factor receptor, ephrin receptor A2 [44], and the cholesterol transporter Niemann-Pick C1-like 1 also participate to the entry process [45]. The particle is internalized by clathrin-mediated endocytosis [46] and the viral genome is released into the cytosol of the cell following the fusion of the viral envelope and the endosomal membrane.

Once in the cytosol, the viral genome is translated. Non-structural viral proteins NS3/4A, NS4B, NS5A, and NS5B assemble into replication complexes [47] that generate new viral genomic RNA molecules through the prior synthesis of negative RNA strands, complementary to the genomic RNA. Much like for many positive stranded RNA viruses, HCV replication occurs in host cell cytoplasm in association with rearranged membranes, named ‘membranous webs’ [48]. A large number of host factors probably participate to the formation and the functioning of HCV replication complexes, which are recruited through interactions with viral proteins. A major host cell factor regulating HCV replication recently identified is the class III phosphatidylinositol 4-kinase alpha [49,50]. The protease NS3/4A and the RNA polymerase NS5B are the two major druggable viral factors involved in HCV replication, which have been used in antiviral screens.

The assembly step of the HCV life cycle occurs in the vicinity of cytoplasmic lipid droplets (LD). The core protein, which is localized on the surface of LD [51], recruits replication complexes through interaction with NS5A [52]. It was recently shown that p7 and NS2 are involved in the assembly step by interacting with E1E2 envelope glycoproteins and non-structural proteins, mainly NS3, and that these interactions are crucial for the formation of assembly sites [53,54,55,56,57]. Host factors critical for HCV assembly include diacylglycerol acyltransferase-1, a triglyceride-synthesizing enzyme required for core trafficking to LD [58], and VLDL secretion machinery [59,60].

For years, the production of HCV in cell culture has been impossible and the search for DAAs was essentially limited to host and viral targets involved in the replication step of the virus. Most of the early screens were performed based on the *in vitro* protease activity of NS3/4A. With the recent introduction of various assays based on the HCVcc system, the search for DAAs has been highly stimulated and can now be performed in the context of a complete HCV life cycle. During the last few years, this led to a substantial increase of reports on natural compounds displaying an anti-HCV activity. The identified molecules belonging to different chemical families are summarized in Table 1, and the different affected steps of the HCV life cycle depicted in Figure 1. In this review, we have chosen to classify these molecules according to the advances in their proof of concept and to their chemical family. 

**Table 1:** Natural molecules with anti-HCV activities tested *in vivo* or in cellular models.

**Active molecule**	**Viral step**	**Putative viral target**	**IC_50 _**(*in vitro*)	**References**
(extracted from)				
**Silymarin/Silibinin**	Entry (fusion)	NS5B polymerase	40-100 µM	[61,62,63,64,65]	
(*Silybum marianum*)	Replication				
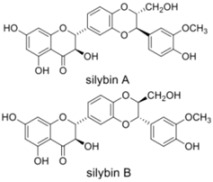	RNA and protein expression				
	Secretion of infectious viral particles				
	Cell-to-cell spread				
		Core protein level	-	[66]	
		HCV RNA	-	[67,68,69,70,71]	
**EGCG**	Early step of entry	HCV virion	5-21 µM	[72,73,74]	
(*Camellia sinensis*)	glycoproteins (attachment)				
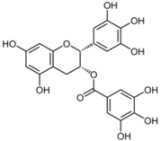	Cell-to-cell spread				
	Clearance of cell culture supernatant				
	Replication	HCV RNA, core, NS3 protease, NS5A		[74]	
**Ladanein-BJ486K**	HCV entry		2.5-10 µM	[75]	
(*Marrubium peregrinum *L.)					
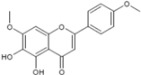					
**Naringenin**	Assembly		109 µM	[76,77]	
(Grapefruit)					
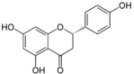	Secretion (core and HCV RNA)				
**Quercetin**	Inhibition of IRES translation	NS3 protease	-	[78,79]	
(*Embelia ribes*)	NS5A protein levels				
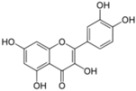	HCV replication				
	HCV production				
**Luteolin-Apigenin**	HCV infection	NS5B polymerase	1.1-7.9 µM	[80]	
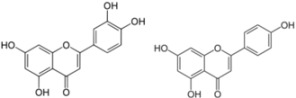	Replication				
**Honokiol**	Entry	NS3 protease, NS5A, NS5B polymerase	4.5 µM	[81]	
*(Magnolia grandiflora)*					
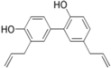	Replication				
**3-hydroxy caruilignan C**	Replication	HCV RNA, NS3 protease	37.5 µM	[82]	
(*Swietenia macrophylla*)					
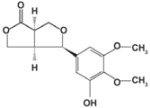					
**Excoecariphenol D**	Replication	NS3 protease	12.6 µM	[83]	
**Corilagin**					
*(Excoecaria agallocha* L.)					
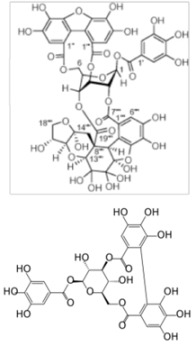					
			13.5 µM		

## 3. Flavonoids

Flavonoids or bioflavonoids are a class of plant secondary metabolites. They are naturally present in numerous plants. More than 4,500 flavonoids have been characterized so far. They have been classified according to their chemical structure and are usually subdivided into different subgroups. Some of them are described as potential anti-HCV molecules. 

### 3.1. Silymarin/Silibinin

Silymarin is extracted from the seeds of milk thistle *Silybum marianum*. This plant native of Southern Europe and Asia is now found throughout the world. The seed extract of milk thistle is an ancient herbal remedy used as hepatoprotectant and to treat liver disease. It contains at least seven flavonolignans (silybin A, silybin B, isosilybin A, isosilybin B, silychristin, isosilychristin, silydianin) and one flavonoid (taxifolin). Flavonolignans are natural polyphenols composed of flavonoid and lignan moieties. The major component of silymarin, silibinin (a mixture of the two diastereoisomers silybin A and silybin B) has also been reported to have anti-HCV activity.

Silymarin has multiple effects on HCV. Silymarin appears to inhibit HCV infection at least at two different levels: it inhibits HCV replication in cell culture [61] and it also displays anti-inflammatory and immunomodulatory actions that may contribute to its hepatoprotective effects [84]. By screening the seven major flavonolignans, Polyak *et al*. showed that specific compounds present in silymarin are responsible for the different anti-HCV activities [61,64]. The inhibition of HCV replication was attributed to the inhibitory action of silibinin on the NS5B RNA-dependent RNA polymerase [63,65]. Half inhibitory concentrations (IC_50_) in the order of 75–100 µM and 40–85 µM were reported in these studies for a succinate-conjugated form of silibinin, which is more soluble in aqueous solutions than natural silibinin. An inhibition of entry was also reported with HCVpp and liposome fusion assays [62,63]. Another potential anti-HCV activity of silymarin has been described by Ashfaq *et al*. [66]. Using a heterologous expression system, they reported an NS5B-independent inhibition of HCV genotype 3a core expression by silymarin. 

Low bioavailability of silymarin components has been reported [85]. This is probably the reason why clinical studies dealing with oral administration of silymarin have been unsuccessful in curing patients from HCV [85,86,87]. Because silibinin is rapidly metabolized after oral administration [88], clinical studies were also attempted with the water-soluble, succinate-conjugated silibinin formulated for intravenous injection. In this case, silibinin monotherapy showed a substantial antiviral effect in patients with chronic hepatitis C not responding to standard pegylated interferon/ribavirin therapy [67,68]. Two cases of successful prevention of liver graft infection with silibinin monotherapy in patients with chronic hepatitis C have also been reported [69,70], and a case of sustained virological response after treatment with intravenous silibinin was reported for a HCV/HIV co-infected patient not responding to the standard HCV therapy [71]. Therefore, although oral administration of silymarin is not effective for the treatment of HCV patients, intravenous silibinin formulation may represent a potential therapeutic option.

### 3.2. (−)-Epigallocatechin-*3*-gallate (EGCG)

EGCG is the most abundant flavonoid from the subclass of catechin present in green tea extract. It has been shown that a single cup of tea contains up to 150 mg of this molecule and its administration is safe in healthy individuals [89]. Very recently, three different groups have independently identified EGCG as a new inhibitor of HCV entry [72,73,74]. These studies showed that EGCG present during infection of Huh-7 cells with HCVcc resulted in dose-dependent inhibition of infection. Different IC_50_ (between 2.5 µg/mL and 9.7 µg/mL, corresponding to 5 µM and 21 µM) were obtained in the three studies, probably at least in part reflecting differences in experimental setups. The half cytotoxic dose was comprised between 150 and 175 µM in Huh-7 cells, depending on the exposure time. Two groups found no additional effect of EGCG on HCV RNA replication and on release of HCV infectious particles [72,73], despite reported inhibitory activities of EGCG on NS3 and NS5B in *in vitro* assays [90,91], while the third group reported an additional activity of EGCG on the RNA replication step [74]. Hepatitis C virus pseudoparticles were used to further confirm the impact of EGCG on HCV entry. EGCG inhibited HCVpp entry in a genotype-independent manner in hepatoma-derived cells [72,73,74], as well as in primary human hepatocytes [72].

The mechanism of action of EGCG on HCV entry is still being investigated. EGCG inhibits HCV entry only when it is present during the inoculation period, or when viral particles have been pre-incubated with it [72,73,74]. In contrast, the pre-incubation of target cells has no impact on HCV infection. Moreover, EGCG does not change the expression levels of cellular entry factors (CD81, CLDN1, OCLN, SR-BI) [72,74]. Therefore it is very likely that EGCG acts directly on the viral particle. EGCG inhibits the binding of the virus to the cell surface [72,73] and has no effect when added post-binding. It does not appear to alter physical properties of HCV virions, such as their density profile or lipoprotein association [72]. Although antiviral activities of EGCG have also been reported against other viruses, such as herpes simplex virus and influenza virus [92,93,94], the antiviral effect of EGCG on HCV cannot be generalized to the other members of the *Flaviviridae* family, because this molecule is inactive against bovine viral diarrhea virus (BVDV, a pestivirus) or yellow fever virus (YFV, a flavivirus) [73]. Based on its action on both HCVcc and HCVpp, it can be speculated that EGCG interacts with E1E2 glycoproteins and that this interaction inhibits virion binding to target cell surface. However a direct experimental evidence for this interaction is still missing.

Interestingly, EGCG could inhibit cell-to-cell transmission [72,73,74] in addition to its action on cell-free particle binding. Cell-to-cell transmission is probably a major route of spreading of HCV in the liver of infected patients. It was also reported that EGCG could be used in combination with boceprevir or cyclosporin A (two known inhibitors of HCV replication), with an increased efficiency [72]. Finally, it was shown that the anti-HCV effect of EGCG can lead to undetectable levels of virions in the supernatant of Huh-7 infected-cells after a few passages [73,74]. Recently, Fukazawa *et al*. [95] by developing a new anti-HCV molecule-screening assay, have confirmed the anti-HCV activity of EGCG. Moreover, consumption of up to 800 mg of EGCG is safe and increases the concentration of EGCG detected in the plasma [89], indicating its potential use in clinical trials. 

All these data indicate that EGCG is a new anti-HCV molecule with interesting properties. It directly inactivates HCV particle, is not genotype-specific (unlike currently used protease inhibitors), and also prevents cell-to-cell transmission. These properties make it an especially interesting molecule to prevent HCV recurrence and spread in chronically infected liver transplant patients. Future clinical trials should investigate whether it could actually prevent the re-infection of patients undergoing orthotopic liver transplantation, and whether it could be used in combination with other DAAs to treat infected patients.

### 3.3. Ladanein

Recently, Haid *et al*. have isolated a molecule with anti-HCV activity in a screen of a library of natural phenolic compounds from plant extracts [75]. From the most active plant extract, they characterized and re-synthesized the component exhibiting the highest antiviral activity. Ladanein (and its synthetic equivalent BJ486K) was identified as the active anti-HCV component. Ladanein, extracted from *Marrubium peregrinum* L. (*Lamiaceae*), is a flavone, a molecule belonging to a subgroup of the flavonoid family. Ladanein inhibited HCV entry with an IC_50_ of 2.5 µM in a genotype-independent manner. In contrast to EGCG, ladanein did not appear to inhibit the binding of the viral particle (although results of a direct binding assay were not reported), but rather inhibited a later, yet uncharacterized step of viral entry. Interestingly, when used in combination with cyclosporin A, a known inhibitor of HCV replication, ladanein acted synergistically on HCV infection. Ladanein also exhibited an antiviral activity in primary human hepatocytes, but with an increased IC_50_ (10 µM). Very importantly, this molecule was shown to be orally bioavailable in mice with a peak of plasma level of 329 nM after a single oral dose of 0.25 mg/kg. These data are encouraging for a potential use of ladanein as an anti-HCV molecule in patients.

### 3.4. Naringenin

Naringenin is a dietary supplement demonstrated to possess anti-oxidant, anti-inflammatory, and anti-carcinogenic properties both *in vitro* and *in vivo*. This molecule belongs to the flavonoid family. It is the predominant flavanone present in the grapefruit and is responsible for its bitter taste. Naringenin has been previously shown to reduce cholesterol levels both *in vitro* [96] and *in vivo* [97]. Furthermore, naringenin inhibits ApoB secretion by reducing the activity and the expression of the microsomal triglyceride transfer protein (MTP) and the acyl-coenzyme A cholesterol acyltransferase 2 (ACAT) [96,98]. Due to the close link between HCV assembly/secretion and lipoprotein metabolism, Nahmias *et al.* have studied the impact of naringenin on the secretion of HCV particles [76]. A concomitant dose-dependent decrease of core protein, HCV-positive strand RNA, infectious particles, and ApoB was observed in the supernatant of infected Huh-7 cells after naringenin treatment [76]. The inhibitory activity of naringenin was also observed in primary hepatocytes in culture. Naringenin blocked the assembly of intracellular infectious viral particles without affecting intracellular levels of the viral RNA or protein. The maximal inhibition (74% of inhibition) of secretion of both ApoB and HCV RNA is observed at 200 µM naringenin with an IC_50_ of 109 µM [77].

The mechanism of action of naringenin was proposed to be through the inhibition of ApoB secretion. In a first study, this inhibition was correlated to a reduction of the activity of the MTP and an inhibition of the transcription of 3-hydroxy-3-methyl-glutaryl-coenzyme reductase (HMGR) and ACAT, three enzymes involved in the production of VLDL [76]. The authors later observed that naringenin also induces peroxisome proliferator-activated receptor alpha (PPARα) and that naringenin inhibition of HCV secretion can be reversed by a PPAR inhibitor [77]. Naringenin caused an increased expression of PPARα and its target gene acyl-CoA oxydase and a concomitant decrease in sterol regulatory element-binding protein (SREBP) and its target HMGR. PPARα induction is known to inhibit cholesterol synthesis through SREBP and its target gene, HMGR. These data suggest that naringenin effect is at least partially mediated by PPARα activation. 

### 3.5. Quercetin

Quercetin is a flavonol, a plant-derived flavonoid, present in fruits, vegetables, leaves and grains. This molecule has been described as an anti-HCV molecule by two different teams. In 2009, Gonzalez *et al*. were looking for novel cellular proteins that interact with the viral protein NS5A (from H77 strain (genotype 1a)) of HCV [78]. By co-immunoprecipitation and co-localisation assays, they detected an interaction between NS5A and the heat shock proteins (HSP) HSP40 and HSP70. In order to confirm the implication of these proteins in the HCV life cycle, they tested the impact of quercetin, a known inhibitor of HSP synthesis. Using a cell culture-based bicistronic reporter system, quercetin was found to decrease IRES activity either in absence or in presence of NS5A. Quercetin also had a strong inhibitory effect at 50 µM on HCV production in cell culture. However, its mechanism of action is not clear, because siRNA-mediated depletion of HSP proteins had no effect on HCV particle production, and because quercetin had only a modest effect on replication in the HCVcc system, and did not inhibit HCV replication in a subgenomic replicon system. Therefore, the anti-HCV action of quercetin could be related to an impairment of viral morphogenesis or secretion, rather than to a direct action on the replication step of the HCV life cycle.

By screening for NS3 inhibitors in traditional Indian medicinal plants, Bachmetov *et al*. recently identified quercetin as the active substance responsible for the inhibition of NS3 protease activity by *Embelia ribes* plant extracts [79]. This inhibition of NS3 was confirmed in cell culture with NS3 protease-dependent fluorescent reporter systems. Again, the authors found a strong inhibition of HCV particle production at the dose of 10 µg/mL (≈33 µM), and a weaker impact on replication.

Unfortunately, in both studies, entry and secretion were not investigated with specific assays in order to know if the effect observed on HCV replication was only due to the single action of this molecule at the replication step or if it results from an additional effect on other steps of the HCV life cycle.

### 3.6. Luteolin and Apigenin

Luteolin and apigenin, two other natural flavone molecules, were identified as anti-HCV agents via a pharmacophore search [80]. A pharmacophore corresponds to a theoretical description of molecular features, which can be used for probing specific interactions between a ligand and a biological molecule. In this study, the designed pharmacophore was established from eight NS5B inhibitors selected from the literature according to different criteria. The resulting pharmacophore was tested against 15,568 compounds from an in-house database. Only 31 compounds were potentially relevant and were evaluated for their anti-HCV activities *in vitro*. Finally, 20 compounds showed a significant activity against HCV (half maximal effective concentration, EC_50_ < 50 µM). Among them, the most potent molecules were luteolin and apigenin. Luteolin and apigenin displayed an anti-HCV activity with EC_50_ values of 4.3 µM and 7.9 µM respectively in a cell-based antiviral assay. Finally, the authors showed that luteolin exhibited a good inhibition of NS5B polymerase enzymatic function with an IC_50_ of 1.12 µM according to the method used [99].

## 4. Lignans

The lignans are a group of chemical compounds found in plants. Lignans are one of the major classes of phytoestrogen, which are estrogen-like chemicals and act as antioxidants.

### 4.1. Honokiol

Honokiol is a lignan present in the cones, the bark and the leaves of *Magnolia officinalis*. This plant has been used in the traditional Japanese medicine Saiboku-to. In 2011, Lan *et al*. showed that honokiol inhibits HCV infection [81]. The effect of honokiol on HCV infection, entry, translation and replication was assessed in Huh-7 cells using HCVcc, HCVpp and subgenomic replicons. Honokiol strongly inhibited HCVcc infection (EC_50_ = 1.2 µg/mL, corresponding to 4.5 µM, and EC_90_ = 6.5 µg/mL) at non-toxic concentrations (median lethal dose = 35 µg/mL). Combined with IFN-α, its inhibitory effect on HCVcc was more profound than that of ribavirin combine with interferon. Honokiol-mediated inhibition of HCV infection was shown to result from multiple effects on the HCV life cycle. Honokiol inhibited the entry of HCVpp from genotypes 1a, 1b and 2a. Honokiol dose-dependently inhibited the expression levels of NS3, NS5A and NS5B. It also inhibited the replication of genotypes 1a and 2a subgenomic replicons in a dose-dependent manner. The authors conclude that the inhibition of both entry and replication by honokiol provides the impetus to fully explore the clinical utility of honokiol as an adjunct to current standards of treatment for HCV infection.

### 4.2. 3-Hydroxy Caruilignan C

In 2012, Wu *et al.* reported the anti-HCV effect of 3-hydroxy caruilignan C (3-HCL-C) isolated from *Swietenia macrophylla* stems [82]. *Swietenia macrophylla* belongs to the *Meliaceae* family and its fruits are used as a folk medicine in Malaysia. 3-HCL-C reduced both protein (NS3) and RNA levels of HCV with an EC_50_ value of 10.5 µg/mL (corresponding to 37.5 µM) in the subgenomic replicon system. Moreover, combinations of 3-HCL-C and IFN-α, 2'-C-methylcytidine (NM-107, an NS5B polymerase inhibitor) or telaprevir (VX-950, an NS3/4A protease inhibitor) increased the suppression of HCV RNA replication. 3-HCL-C interfered with HCV replication by inducing IFN-stimulated response element transcription and IFN-dependent anti-viral gene expression. Therefore, 3-HCL-C has the potential to be developed into a potent adjuvant for anti-HCV therapy.

## 5. Other Polyphenols

Other polyphenols have been reported to have potential anti-HCV activity using *in vitro* assays. Suzuki *et al*. identified 3',4',5,6,7,8-hexamethoxyflavone, also known as nobiletin, as the active compound responsible for the anti-HCV activity of Citrus unshiu peel (*Aurantii nobilis* pericarpium) extract, an ingredient of traditional Japanese Kampo medicine [100]. Nobiletin displayed an anti-HCV activity at 10 µg/mL in MOLT-4 cells infection assay. Hegde *et al*. isolated and characterized two novel oligophenolic compounds, named SCH 644343, and SCH 644342 from the Peruvian plant *Stylogne cauliflora* [101]. These two compounds were identified as inhibitors of HCV NS3 protease activity *in vitro* with IC_50_ of 0.3 µM and 0.8 µM respectively. SCH 644343 was also active in an NS3 binding assay (IC_50_ = 2.8 µM). Zuo *et al.* identified the 1,2,3,4,6-penta-*O*-galloyl-β-D-glucoside as a potent inhibitor of NS3 protease activity from the plant *Saxifraga melanocentra* Franch [102]. The IC_50_ was 0.68 µM and the molecule was not toxic up to 6 mg/mL (corresponding to 6.4 mM) on COS cells. Duan *et al*. identified three polyphenol components from the ethyl acetate fraction of the traditional Chinese medicine *Galla Chinese* [103]. These polyphenols molecules, 1,2,6-tri-*O*-galloyl-β-D-glucose, 1,2,3,6-tetra-*O*-galloyl-β-D-glucose and 1,2,3,4,6-penta-*O*-galloyl-β-D-glucose, were shown to inhibit NS3 protease *in vitro* with IC_50_ values of 1.89, 0.75 and 1.60 µM, respectively.

All these compounds were identified before the HCVcc model was established and to our knowledge, they have not been evaluated since then. Therefore, they will need to be tested in cell-based assays, before considering them as new potential anti-HCV agents. This is illustrated by the study of Li *et al*. who identified four polyphenolic compounds inhibiting NS3 protease *in vitro*, from the Chinese mangrove plant *Excoecaria agallocha* L. (*Euphorbiaceae*) [83]. Among them, only two, excoecariphenol D and corilagin had a significant inhibitory action in the replicon assay with an IC_50_ of 12.6 and 13.5 µM respectively. 

## 6. Crude Plant Extracts from Traditional Medicines

Traditional medicines represent a broad source of natural polyphenols molecules. In 2003, Liu *et al*. assessed beneficial and harmful effects of medicinal herbs against HCV infection. Thirteen randomized trials have evaluated fourteen medicinal herbs. Only four trials had an adequate evaluation method [104]. Even if traditional medicine represents an attractive source of new natural antivirals, studies with herbs need to be standardized in order to clearly evaluate the effects due to the plant extracts on HCV infection, and should provide all methodological details. Compounds isolated from these herbs could be used for designing and developing drugs for treatment of hepatitis C.

A study, which evaluated the benefice of a Far-Eastern traditional herbal formulation for patients with chronic hepatitis C [105], found some improvement of circulating aminotransferases, with no effect on HCV RNA levels. Several other studies evaluated the effect of plant extracts from traditional medicine using various assays, which found extracts with anti-NS3 protease activity [106,107], anti-NS5B activity [16,107] and with anti-replicative effect in the replicon model [107,108,109]. None of these studies tried to evaluate other steps of the HCV life cycle. In two studies, additive or synergistic actions in combination with interferon [107,108], telaprevir or 2'-C-methylcytidine [108,109] were reported. Future studies on these plant extracts may lead to the identification of new anti-HCV molecules.

## 7. Concluding Remarks

In this review, we discussed the diverse and broad actions of natural molecules issued from plants as potential anti-HCV antivirals. It is important to note that some other natural compounds, even if they do not target the virus directly, might also be used to improve HCV therapy. Glycyrrhizin, for instance, a component of licorice roots extract, has been shown to prevent the development of hepatocellular carcinoma in patients with chronic hepatitis [110].

Although a number of natural compounds with anti-HCV activities were identified in recent years, many aspects concerning their mechanisms of action remain unknown. Very often, the replication was the only step of the viral life cycle that was investigated and, for older reports, the conclusions are only based on *in vitro* models, mostly NS3 protease assays. Yet, ladanein and EGCG proved to be potent entry inhibitors, and reports on quercetin anti-HCV activity suggest that it may in fact be more active on the assembly, than on the replication step. The HCVcc model now allows to identify anti-HCV molecules in the context of a complete life cycle and then to pinpoint the inhibited step, with additional and more specific assays. Importantly, we should keep in mind that the *in vitro* inhibition of the enzymatic activity of a viral protein by a natural compound does not conclusively demonstrate that this viral protein is the *bona fide* target of the compound. Additional binding and crystallization studies are required to prove the point. For example, in the case of silymarin/silibinin, high concentrations are required to inhibit NS5B *in vitro*. It is possible instead that a cellular protein target could mechanistically be involved in the antiviral action of these compounds. More investigations are clearly needed to validate numbers of these molecules *in vivo*. As illustrated by silymarin, the bioavailability is an important point to consider, even for molecules extracted from ancient herbal medicines. In conclusion, even if we do not expect that natural molecules may replace the current anti-HCV therapy, treatments could be more likely supplemented, and perhaps lightened by adapted diet, limiting their cost.
